# Supplementary material for: Probing the Effects of the FGFR-Inhibitor Derazantinib on Vascular Development in Zebrafish Embryos
Source: Pharmaceuticals (Basel). 2020 Dec 30;14(1):25. doi: 10.3390/ph14010025 (PMC7824571; doi:10.3390/ph14010025)
Supplement: Supplementary file 1 [file pharmaceuticals-14-00025-s001.zip › Supplementary-Proofs/Pharmaceuticals-Kotini et al-2020-Supplementary-Revised-Proof.docx]

Supplementary Materials

Kotini et al. “Probing the effects of the FGFR-inhibitor Derazantinib on vascular development in zebrafish embryos”

Supplementary Figures

**Figure S1 (related to Figure 1). *Derazantinib* inhibits vascular development *in vivo* in a dose dependent manner.** Confocal images of GFP+ blood vessels in the trunk of *Tg(fli1:EGFP)^y1^* zebrafish embryos at 26 hpf (**a-e**) or 45hpf (**f-j**) after exposure to increasing concentrations of DZB in the swimming water. Blood vessel development appears similar to control using concentrations between 0.001- 0.03 µM DZB and was disrupted using 10 µM concentration of DZB. Asterisks mark sprouting defects. Scale bar, 50µm.

**Figure S2 (related to Figure 3). *Derazantinib* perturbs vascular cohesion in arterial and venous branches.** Confocal images of GFP+ blood vessels and RFP+ arteries in the trunk of *Tg(fli1:EGFP)^y1^/Tg(-0.8flt1:RFP)^hu5333^* transgenic embryos from 45 hpf in control (DMSO) or treated embryos with INF(**b**) or VAT(**c**) or DZB (**d-e**). DZB (**b, d**). Asterisks mark the absence of blood vessel spouts in the VAT treatment. Artery to vein ratio appeared similar between control and treated embryos (**f**). Treatment with DZB or INF leads to blood vessel disconnections (arrowheads) at the DLAV or at the dorsal aorta (DA) or posterior cardinal vein (PCV) and thinning of blood vessels (arrows) compared to control (**a-c**). (**f**) Quantitative analysis of ratio of arterial and venous sprouts per zebrafish embryo (n≥10 embryos per treatment were analysed from 3 independent experiments) and quantitative analysis of ISV sprouts that are disconnected at the DLAV (normalised to total number of DLAV branches) or at the DA/ PCV (normalised to total number of ISVs) per embryo (n≥10 embryos per treatment were analysed from 3 independent experiments). Although the artery/vein ratio is similar between treatments and control group (DMSO), DZB treatment interferes much more with arterial vessel disconnections at the DLAV and venous vessel disconnections at the DA or PCV. Data in **f** represent mean ± S.E.M. (error bars), ns: not significant, *P<0.05 **P<0.01, ***P<0.001. Statistical analysis was performed with the two-sided Mann–Whitney test. Scale bar, 50µm.

**Figure S3 (related to Figure 4). *Derazantinib*** **and** ***infigratinib* interfere with endothelial cell junctions.** Confocal images of GFP+ endothelial cell junctions and mCherry+ endothelial cell membranes in *Tg(fli1:pecam1-EGFP)^ncv27^*/*Tg*(*kdrl:mCherry-CAAX*)*^s916^* transgenic embryos from 40 hpf after treatment with DMSO (control, **a-a’**), DZB (**b-b’**), INF (**c-c’**) or VAT (**d-d’**). **a’-d’** depict only the GFP+ channel. Arrowheads point to multicellular continuous cell junctions, indicative in the control group (DMSO, a-a’), while arrows point to linear discontinuous junctions, indicative in the DZB-treated embryos (**b-b’**) and INF-treated embryos (**c-c’**). Asterisks mark lack of blood vessel sprouting (**d-d’**). Scale bar, 50µm.

**Supplementary Figure 4 (related to Figure 5). *Derazantinib* and *infigratinib* inhibit endothelial cell cycle.** Time-lapse images of sprouting ISVs of GFP+ endothelial cell nuclei and mCherry+ endothelial cell membranes in *Tg*(*kdrl:EGFP-nls*)*^ubs1^*/*Tg*(*kdrl:mCherry-CAAX*)*^s916^* transgenic embryos from 26 hpf after treatment with DMSO (**a-a’’’**) or 1uM DZB (**b-b’’’**) or 0.1uM INF(**c-c’’’**). Numbers indicate cell nuclei (ie.1, 2, 3, ...) or cell nuclei arising after cell division (ie. 1.1, 1.2, ...). Scale bar, 50µm.

Supplementary Videos

**Video 1 (related to Figure 2). Blood flow in DMSO-treated embryo.** Time-lapse confocal imaging of GFP+ blood vessels and DsRed+ erythrocytes in a *Tg(fli1:EGFP)^y1^/Tg(gata1:DsRed^)sd2^* representative zebrafish embryos at 36hpf after exposure to DMSO as control. Scale bars, 50µm.

**Video 2 (related to Figure 2). Blood flow in DZB-treated embryo.** Time-lapse confocal imaging of GFP+ blood vessels and DsRed+ erythrocytes in a *Tg(fli1:EGFP)^y1^/Tg(gata1:DsRed^)sd2^* representative zebrafish embryos at 36hpf after exposure to 3 μM *derazantinib*. Scale bars, 50µm.

**Video 3. (related to Figure 5a). Angiogenic sprouting and cell divisions in DMSO-treated embryos.** Time-lapse confocal imaging of sprouting ISVs of GFP+ endothelial cell nuclei and mCherry+ endothelial cell membranes in a *Tg*(*kdrl:EGFP-nls*)*^ubs1^*/*Tg*(*kdrl:mCherry-CAAX*)*^s916^*  representative embryo from 26 hpf after treatment with. Scale bars, 20µm.

**Video 4. (related to Figure 5b). Angiogenic sprouting and cell divisions in 0.3 µM** **DZB-treated embryos.** Time-lapse confocal imaging of sprouting ISVs of GFP+ endothelial cell nuclei and mCherry+ endothelial cell membranes in a *Tg*(*kdrl:EGFP-nls*)*^ubs1^*/*Tg*(*kdrl:mCherry-CAAX*)*^s916^*  representative embryo from 26 hpf after treatment with 0.3 µm DZB. Scale bars, 20µm.

**Video 5. (related to Figure 5c). Angiogenic sprouting and cell divisions in 3 µM** **DZB-treated embryos.** Time-lapse confocal imaging of sprouting ISVs of GFP+ endothelial cell nuclei and mCherry+ endothelial cell membranes in a *Tg*(*kdrl:EGFP-nls*)*^ubs1^*/*Tg*(*kdrl:mCherry-CAAX*)*^s916^*  representative embryo from 26 hpf after treatment with 3 µm DZB. Scale bars, 20µm.

**Video 6. (related to Figure 5d). Angiogenic sprouting and cell divisions in 0.3 µM** **INF-treated embryos.** Time-lapse confocal imaging of sprouting ISVs of GFP+ endothelial cell nuclei and mCherry+ endothelial cell membranes in a *Tg*(*kdrl:EGFP-nls*)*^ubs1^*/*Tg*(*kdrl:mCherry-CAAX*)*^s916^*  representative embryo from 26 hpf after treatment with 0.3 µm INF. Scale bars, 20µm.
